# Pharmacokinetics and tissue distribution of bleomycin-induced idiopathic pulmonary fibrosis rats treated with cryptotanshinone

**DOI:** 10.3389/fphar.2023.1127219

**Published:** 2023-03-09

**Authors:** Xiangjun He, Zhi Zhong, Quan Wang, Zhenmao Jia, Jing Lu, Jianwen Chen, Peiqing Liu

**Affiliations:** ^1^ National and Local United Engineering Lab of Druggability and New Drugs Evaluation, School of Pharmaceutical Sciences, Sun Yat-Sen University, Guangzhou, China; ^2^ Guangdong Provincial Key Laboratory of New Drug Design and Evaluation, School of Pharmaceutical Sciences, Sun Yat-Sen University, Guangzhou, China

**Keywords:** cryptotanshinone, pulmonary fibrosis, comparative pharmacokinetic, tissue distribution, HPLC-MS/MS

## Abstract

**Introduction:** Cryptotanshinone(CTS), a compound derived from the root of *Salvia miltiorrhiza*, has been linked to various of diseases, particularly pulmonary fibrosis. In the current study, we investigated the benefit of CTS on Sprague-Dawley (SD) rats induced by bleomycin (BLM) and established high performance liquid chromatography-tandem mass spectrometry (HPLC-MS/MS) methods to compare pharmacokinetics and tissue distribution in subsequent normal and modulated SD rats.

**Methods:** The therapeutic effect of CTS on BLM-induced SD rats was evaluated using histopathology, lung function and hydroxyproline content measurement, revealing that CTS significantly improved SD rats induced by BLM. Additionally, a simple, rapid, sensitive and specific HPLC-MS/MS method was developed to determine the pharmacokinetics of various components in rat plasma.

**Results:** Pharmacokinetic studies indicated that CTS was slowly absorbed by oral administration and had low bioavailability and a slow clearance rate. The elimination of pulmonary fibrosis in 28-day rats was slowed down, and the area under the curve was increased compared to the control group. Long-term oral administration of CTS did not accumulate *in vivo*, but the clearance was slowed down, and the steady-state blood concentration was increased. The tissue distribution study revealed that CTS exposure in the lungs and liver.

**Discussion:** The lung CTS exposure was significantly higher in the model group than in the control group, suggesting that the pathological changes of pulmonary fibrosis were conducive to the lung exposure of CTS and served as the target organ of CTS.

## 1 Introduction

Pulmonary Fibrosis (PF) is a chronic, progressive and irreversible lung disease common in clinical practice. In the early stage, it is characterized by alveolar epithelial cell injury, interstitial lung inflammation and interstitial lung edema. In the end stage, a large amount of Extracellular Matrix (ECM) deposition, abnormal proliferation, activation of fibroblasts and destruction of tissue structure destruction occur (Urban et al., 2015; Barratt et al., 2018; Sgalla et al., 2018). The lung tissue thickens, scar tissue forms and lung function decrease significantly, eventually developing organ dysfunction and respiratory failure (King et al., 2011). Pulmonary fibrosis is prevalent in the elderly, and its incidence increases yearly. The average life expectancy after diagnosis is approximately 2.8 years, and the salvage rate is lower than for most tumors (Chanda et al., 2019). The etiology of pulmonary fibrosis is complex. Many factors are known to cause pulmonary fibrosis, such as smoking, environmental pollution, lung injury, virus and drugs (Rangarajan et al., 2016). Idiopathic pulmonary fibrosis (IPF), the most severe form of pulmonary fibrosis, has a high mortality rate and a poor prognosis (Richeldi et al., 2017). Pulmonary fibrosis is treated primarily with glucocorticoids, anti-inflammatory drugs, immunosuppressants, and antifibrotic drugs. Although drug therapy can alleviate disease symptoms and improve respiratory function, long-term use is prone to adverse reactions (du Bois, 2010) and cannot significantly improve the survival rate of patients (Spagnolo et al., 2015). It is important to obtain more effective drugs due to adverse reactions and the limited effectiveness of existing drugs in preventing and treating fibrosis. Additionally, recent studies (Li et al., 2022) reveal that various natural small-molecule compounds have certain therapeutic effects on pulmonary fibrosis, so developing natural small-molecule compounds is important.

Cryptotanshinone (CTS) is a diterpenoid quinone lipid-soluble compound extracted from the root of *S. miltiorrhiza*. It benefits from abundant sources, low toxicity and low relative molecular weight. It has high biological activity and high content among the extracts of *S. miltiorrhiza*. Recently, CTS has been proven to have anti-inflammatory, antioxidant, anti-angiogenic and anti-proliferative activities, and play a role in various malignant tumors (Wang et al., 2017; Qi et al., 2019; Chen et al., 2020; Luo et al., 2020), cardiovascular diseases (Zhang et al., 2021), neuroprotection (Kwon et al., 2020) and other diseases. Our laboratory has conducted multiple studies on CTS, and discovered that CTS could significantly improve pulmonary fibrosis in rats induced by bleomycin (BLM), and reverse the fibrosis level of human fetal lung fibroblasts (HLF) induced by factor-beta 1 (TGF-β1) by inhibiting the STAT3 and Smad2/3 phosphorylation (Zhang et al., 2019). Furthermore, our laboratory revealed that treatment with CTS attenuates adult rat cardiac fibroblasts and cardiac fibrosis rats induced by angiotensin Ⅱ (Ma et al., 2014). However, the comparative pharmacokinetics and tissue distribution of CTS under normal and model conditions remain unclear.

Pharmacokinetic (PK) based studies are considered a reliable approach for identifying and screening potential bioactive components that contribute to the pharmacological effects of natural compounds and to better elucidate their mechanisms of action (Sun et al., 2012). Numerous factors, including species, age, sex, mode of administration, dose of administration, and disease (Labrecque and Bélanger, 1991; Lin, 1995; Meibohm et al., 2002; Shi and Klotz, 2011), affect drug absorption (A), distribution (D), metabolism (M), and excretion (E). Diabetes (Pass et al., 2002; Wang et al., 2003; Lam et al., 2010), liver injury (Adawi et al., 2007; Li et al., 2010), chronic heart failure (Huang et al., 2021), inflammatory diseases (Gong et al., 2009; Cressman et al., 2012) and fever (Gao et al., 2014) may cause significant changes in the body’s drug metabolic enzymes, transporters, cell permeability and intestinal microbiota, affecting the ADME process of drugs. Therefore, studying animal or human pharmacokinetic parameters under pathophysiological and normal conditions may help us better understand the mechanism of pharmacodynamic action. According to pharmacokinetic studies, CTS is widely distributed in fat and mucosal tissues, accumulating most in rat lungs after oral or intravenous injection (Pan et al., 2008).

This study examines the pharmacokinetics of A in rats using the LC-MS/MS method established by Song et al. (2005). This method has the advantages of sensitivity and high efficiency. It plays an important role in a pharmacokinetic study. This study may promote the CTS for the first time based on a study of the pharmacodynamics of rats with pulmonary fibrosis and normal rats lavage for drug pharmacokinetics and reveal the CTS in the dynamic change law of pulmonary fibrosis in rats *in vivo*. Tissue distribution study may discuss the distribution of the CTS in the body, and lung targeting intends to elucidate the relationship between distribution and pharmacodynamics *in vivo*.

## 2 Materials and methods

### 2.1 Reagents and chemicals

Cryptotanshinone standard (HPLC) was purchased from Aladdin, and a Loratadine (LTD) standard (HPLC) from China Institute for Pharmaceutical and Biological Products. Methanol (HPLC) was acquired from Amethyst. Ethyl acetate (HPLC), purchased from Kermel and formic acid (HPLC) from Aladdin. Ultrapure water was attained from made by laboratory, Bleomycin from Macklin, Hydroxyproline test box from Nanjing Jiancheng Bioengineering Institute and sodium carboxymethylcellulose cellulose from Tianjin Zhiyuan Chemical Reagent Co., LTD., Normal saline was attained from Jiangxi Kelun Pharmaceutical Co., LTD., Sodium pentobarbital, purchased from Beijing Huayye Huanyu Chemical Co., LTD. and 4% paraformaldehyde from Sevier Bio.

(1) Bleomycin sulfate solution: The molding dose was 5 mg/kg, and the volume of trachea infusion was 0.1 mL/100 g. The concentration of bleomycin solution prepared with normal saline was 5 mg/mL.
(2) Sodium carboxymethyl cellulose slurry: Weigh 5.0 g sodium carboxymethyl cellulose powder, sprinkle it in a beaker containing 1,000 mL distilled water, stir well, and place it overnight, make it fully expanded, get 0.5% sodium carboxymethyl cellulose slurry.
(3) Cryptotanshinone suspension: The administration dose was 60 mg/kg, and the administration volume was 1 mL/100 g by gavage, that is, the concentration of prepared cryptotanshinone suspension was 6 mg/mL. The powder was fully ultrasonic to form a stable suspension for use.
(4) 1% sodium pentobarbital: The anesthetic dose of rats was 45 mg/kg and intraperitoneally injected. Check whether any crystals precipitate before use. Heat it in a 37°C water bath to dissolve it completely.

### 2.2 Animals and treatments

This study followed the Guide for the Care and Use of Laboratory Animals (NIH Publication No. 85-23, revised 1996). Specific Pathogen Free (SPF) male Sprague-Dawley (SD) rats weighing 220–260 g were provided and raised in an SPF environment in by the Animal Experiment Center of Sun Yat-sen University East Campus (license number: SCXK 2011-0029). Animal quarantine observation was 3–5 days. The animals’ appearance and physical signs, behavioral activities, body weight, diet and other indicators were observed during this period. Animals in good condition with no abnormal behavior and activity can be tested.

#### 2.2.1 Model establishment and evaluation

Thirty male SD rats were randomly divided into six groups (*n* = 5 rats per group), A to F: (A) 14-day control group; (B) 14-day model group; (C) 28-day control group; (D) 28-day model group; (E) 28-day control group; and (F) 28-day model group. Pulmonary fibrosis was induced through the tracheal infusion of 5 mg/kg bleomycin in the model group, but not in the control group. E and F were administrated 60 mg/kg CTS from the second day after modeling for 28 days, and other groups were not administrated CTS. Lung function and pathology tests were performed in groups A and B on the 15th day after modeling, and in group C-F on the 29th day after modeling.

#### 2.2.2 Single-dose pharmacokinetic study

Twenty male SD rats were randomly divided according to body weight into four groups (*n* = 5 rats per group), 1 to 4: (1) 14-day control group; (2) 14-day model group; (3) 28-day control group; (4) 28-day model group. The rat model of pulmonary fibrosis induced by tracheal infusion of bleomycin was established in the model group, while the control group was not interfered. The 14-day group rats underwent blood were collected at 0.25, 0.5, 1, 1.5, 2, 3, 4, 6, 8, 12, and 24 h after administration CTS on the 14th day after modeling. The 28-day group rats underwent blood were collected at 0.25, 0.5, 1, 1.5, 2, 3, 4, 6, 8, 12, and 24 h after administration CTS on the 28th day after modeling.

#### 2.2.3 Multi-dose pharmacokinetic study

Ten male SD rats were randomly divided into two groups according to body weight (*n* = 5 rats per group): (5) multi-dose control group and (6) multi-dose model group. The model group was induced pulmonary fibrosis by tracheal drip bleomycin, while the control group did not interfere. The rats in the two groups were administrated 60 mg/kg CTS by gavage on the second day after modeling for 28 days. Blood were collected at the 26th and 27th day and 0, 0.25, 0.5, 1, 1.5, 2, 3, 4, 6, 8, 12, and 24 h on the 28th day after modeling after administration.

#### 2.2.4 Tissue distribution study

Forty male SD rats were randomly divided into two groups according to body weight (*n* = 20 rats per group): (7) control group and (8) model group. The model group was induced pulmonary fibrosis *via* tracheal drip bleomycin, while the control group did not interfere. After 28 days of modeling, they were randomly divided into four subgroups according to body weight: 0.5 h group, 3 h group, 10 h group and 24 h group. At 0.5, 3, 10, and 24 h after administration on the 28th day after modeling, the corresponding subgroups of heart, liver, spleen, lung, kidney, and brain tissues were collected separately.

### 2.3 Pulmonary function assay

Lung function indicators of each rat were collected using a small animal pulmonary function instrument (EMKA, France) on the 14th day after modeling for groups A and B and on the 28th day after modeling for groups C–F. The rats were placed into the whole body plethysmography system, and the environment was kept quiet and the ambient air flow rate was stable. After the rats had reached a state of calm, data were collected continuously for more than 5 min. The indexes of lung function can be obtained: inspiratory time, expiratory time, relaxation time, maximum inspiratory volume, maximum expiratory volume, ventilatory volume per min, respiratory rate, end-inspiratory apnea, end-expiratory apnea, and mid-expiratory flow rate.

### 2.4 Histology and morphological analysis

SD rats in groups A and B were sacrificed on the 14th day after modeling, and rats in groups C–F were sacrificed on the 28th day after modeling; their whole lungs of rats were quickly removed. Immediately fixed with 4% paraformaldehyde for 24 h, left lung tissue embedded paraffin and cut into slices of 5 μm. Sections were stained with hematoxylin and eosin (HE), and Masson’s trichrome and lung histopathological changes were evaluated. Sections were photographed using a light microscope (EVOS FL Auto Cell Imaging System, United States).

### 2.5 Measurement of hydroxyproline (HYP) assay

HYP, a unique distribution in connective tissue collagen, is a post-translational product of proline hydroxylation. The hydroxyproline content reflects collagen metabolism and regulation. In this study, HYP content in lung tissue was determined using alkaline hydrolysis (da Silva et al., 2015). Fresh right lung tissue (weight of 80–100 mg) was chopped *in vitro*, add 1 mL of hydrolyzed was added to a test tube. After cooling the tube to room temperature with tap water, the pH of the lysate was adjusted to 6.0–6.8 and 10 mL of double distilled water was added. Then, centrifuged at 3,500 rpm for 10 min, and suck on 1 mL to new test tubes. The following steps of HYP test Kit instructions (Nanjing Jianchen Bioengineering Institute, China, #A030-2) were followed. Each sample was measured at 550 nm using the following formula to calculate HYP content:

$$Hydroxprolinecontent\;\left(\mu g/mg\right)=\frac{Measured\;OD\;value-Blank\;OD\;value}{Standard\;OD\;value-Blank\;OD\;value}\times Standard\;content\times \frac{Lysate\;total\;volume\;\left(mL\right)}{Organization\;wet\;weight\left(mg\right)}$$

### 2.6 Sample processing

The whole blood was extracted quickly from SD rats through main abdominal vein, and the blood was placed in the heparinized collection vessel, centrifuged at 3,000 rpm for 10 min, and the upper plasma was obtained. Precise measurement of 100 uL plasma sample, add 10 μL of loratadine working solution (plasma, heart, spleen, kidney, brain samples use 200 ng/mL LTD working solution; liver and lung samples use 500 ng/mL LTD working solution) and 500 μL ethyl acetate for liquid-liquid extraction. The mixture was vortexed for 1 min and centrifuged at 12,000 × g for 4 min. The organic phase of the upper layer was 400 μL and dried in a vacuum for 2 h at room temperature. The mobile phase [100 μL; methanol-1% formic acid water (90:10, V/V)] was added to redissolve, vortex for 1 min, and centrifuged at 12,000 × g for 3 min at low temperature. Add 100 μL mobile phase [methanol-1% formic acid water (90:10, V/V)] to redissolve, vortex for 1 min, and centrifuge at 12,000 × g for 3 min at low temperature. An aliquot (80 μL) of the supernatant was transferred into a sample vial for HPLC-MS/MS analysis.

### 2.7 HPLC-MS/MS conditions

The injection volume was 5 μL and the flow rate was kept at 0.2 mL/min. The separation was performed using a HyPURITY C18 (I.D. 2.1 mm × 50 mm, 3 μm, Thermo Scientific, US) column. The mobile phase consisted of methanol and water with 1% formic acid (90:10, V/V). The column temperature was 30°C.

Liquid chromatography-mass spectrometry (Thermo Finnigan, TSQ Quantum) was used to detect CTS in biological samples. The sub-source was electrospray ionization (ESI) with positive ion scanning mode, and the scanning mode was Selected Reaction Monitor (SRM). Spray voltage: 4,000 V; Sheath gas: 35 psi; Auxiliary gas: 10 psi; Capillary temperature: 350°C; Peak width of color filter: 20.0 s; Collision gas pressure: 1.9 mtorr; Scanning width: 0.7 m/z; Scanning time: 0.1 s. Our optimized SRM parameters for the analyte and internal standards (ISs) detection are shown in Table 1.

**TABLE 1:** Ionic reaction pairs and collision energies of cryptotanshinone and loratadine.

Compounds	Precursor ion (m/z)	Product ion (m/z)	Mode	CE (V)
Cryptotanshinone	297	251	Positive	21
Loratadine	383	266	Positive	31

### 2.8 Preparation of stock solutions, working solutions, and quality control samples

CTS and LTD standard substances were precisely weighed and dissolved in 50% methanol to prepare 1 mg/mL CTS and LTD reserve solutions. The working solution was marked into blank rat plasma to generate calibration curves, and the following concentrations were obtained:

(1) The first set (for plasma, heart, spleen, kidney and brain samples): the CTS reserve solution was diluted step by step with 50% methanol to obtain the CTS standard curve working solution with the concentration of 10–2,000 ng/mL. CTS Quality control (QC) samples were prepared at low, middle and high concentrations of 20, 200, and 2,000 ng/mL, respectively.

The LTD reserve liquid was diluted step by step with 50% methanol to obtain 200 ng/mL LTD internal standard working liquid.

(2) The second set (for liver and lung samples): the CTS reserve solution was diluted with 50% methanol step by step to obtain the CTS standard curve working solution with a the concentration of 20–5,000 ng/mL. CTS QC samples were prepared at low, middle and high concentrations of 50, 500, and 3,000 ng/mL, respectively.

The LTD reserve liquid was diluted step by step with 50% methanol to obtain 500 ng/mL LTD internal standard working liquid.

All samples were stored at 4°C before UPLC-MS/MS analysis.

### 2.9 Method validation

The developed HPLC-MS/MS method was validated in terms of specificity, linearity, lower limit of quantification, precision, and accuracy.

Specificity was assessed by comparing chromatograms of drug-free blank samples, low-concentration CTS quality control samples, and biological samples treated with 60 mg/kg CTS. The weighted least square regression method was used for linear regression analysis. The horizontal coordinate was the concentration of CTS drug (ng/mL), and the vertical coordinate was the ratio of chromatographic peak area between CTS and LTD. Intraday accuracy and precision were assessed progressively using five replicates of the high, medium, and low QC samples over a single day, whereas intraday accuracy and precision were assessed using five replicates over three consecutive days. The lower limit of quantitation was assessed by substituting the peak area ratio of CTS and LTD into the standard curve and comparing it with the standard concentration.

### 2.10 Pharmacokinetic data and statistical analysis

The experimental data were statistically processed using GraphPad Prism 9.0 biostatistics software (GraphPad Prism 9.0, San Diego, CA, United States). The date was analyzed using one-way Analysis of Variance (ANOVA) combined with Dunnett’s multiple comparison method. The pharmacokinetic software DAS 2.0 was used to calculate the main pharmacokinetic parameters according to the non-av model: the calculated parameters were the maximum plasma concentration (C_max_), elimination half-life (t1/2), time to reach maximum plasma concentration (T_max_), area under the plasma concentration curve (AUC) versus time (AUC_0−t_) from time zero to the time of last measured concentration, AUC from time zero to infinity (AUC_0−∞_), and total body clearance (CL), mean residence time (MRT), Apparent clearance rate (CL/F), Apparent distribution volume (V_d_/F), Mean drug concentration at steady state (Cav), Steady-state plasma concentration (AUC_ss_) and degree of fluctuation (DF).

## 3 Results

### 3.1 Therapeutic effects of CTS on BLM-induced SD rats

**FIGURE 1:**
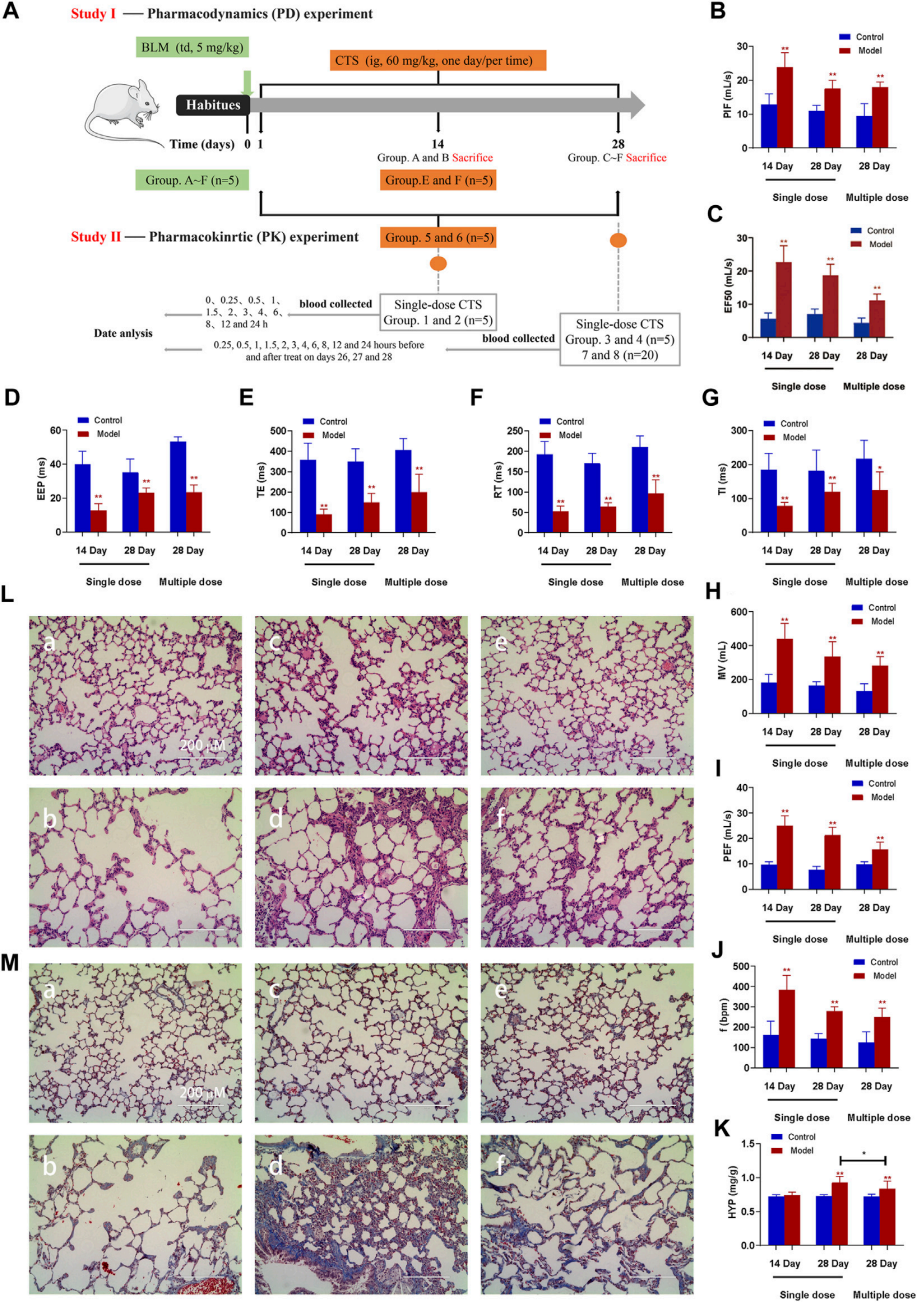
Effect of CTS treatment on BLM-induced SD rats. **(A)** Animal Experiment Diagram for Pharmacodynamic Study (Study Ⅰ) and Animal experiment diagram for pharmacokinetic Study (Study Ⅱ). **(B–J)** Measurement of pulmonary function parameters. **(K)** Determination of hydroxyproline in lung tissue. **(L,M)** H&E and Masson staining of heart tissue in each group; a: 14-day control group; b: 14-day model group; c: 28-day control group; d: 28-day model group; e: control group after 28 days of CTS administration; f: Model group after 28 days of CTS administration; Scale bar = 200 µm. One-way analysis of variance combined with Dunnett’s multiple comparison method was used for analysis, and all data were expressed as mean ± SD. *p < 0.05; **p < 0.01.

Pharmacokinetic studies in model animals are based on the assumption that the animal models are stable and that the pathological changes are consistent with clinical disease characteristics. Endotracheal administration of a single dose of BLM (5 mg/kg) resulted in decreased lung function, implying successful induction of pulmonary fibrosis in SD rats. The maximum inspiratory capacity (PIF, Figure 1B), mid-expiratory flow rate (EF50, Figure 1C), ventilation volume per minute (MV, Figure 1H), maximum expiratory volume (PEF, Figure 1I) and respiratory rate (f, Figure 1I) of rats in each model group were significantly increased. End expiratory apnea (EEP, Figure 1D), expiratory time (TE, Figure 1E), relaxation time (RT, Figure 1F) and inspiratory time (TI, Figure 1G) were significantly reduced compared to the corresponding control group. Nevertheless, the variation trend of all model groups is consistent (Figures 1A–J). The 14-day model group displayed a significant change, followed by the 28-day model group, while the 28-day model group revealed the least change.

At 14 days after BLM treatment, the pulmonary morphology and structure were obviously disordered, with numerous pulmonary bullae and increased infiltration of inflammatory cells, but the pulmonary tissue fibrosis was mild (Figures 1K, L). After 28 days of BLM treatment, numerous collagen fibers appeared in lung tissue, and the thickness of the fibrous scar increased, and the degree of fibrosis aggravated (Figures 1K, L). All indicators were remission in the rat model group treated with CTS for 28 days (Figures 1K, L). Additionally, consistent with MASSON staining results, HYP did not significantly increase after 14 days of BLM treatment, but significantly increased after 28 days. The HYP level was significantly downregulated after CTS treatment (Figure 1M). These results indicated that severe inflammatory reactions destroyed the alveolar structure and seriously affected the respiratory function of rats in the early stage of the model. The alveolar structure was repaired and respiratory function improved with the self-repair of lung tissue. However, the abnormal repair resulted in the production of many collagen fibers and irreversible structural changes in lung tissue. In conclusion, we have successfully constructed pulmonary fibrosis SD rats induced by BLM, and continuous treatment with CTS has a good therapeutic effect on the pathological changes of pulmonary fibrosis SD rats induced by BLM.

### 3.2 HPLC-MS/MS method optimization

Different chromatographic columns were tested to develop an efficient HPLC-MS/MS method for quantitative analysis of CTS. The HyPURITY C18 (I.D. 2.1 mm × 50 mm, 3 μm, Thermo Scientific, US) was compared to Hypersil BDS C18 (I.D. 2.1 mm × 150 mm, 5 μm, Elite HPLC, Dalian, China). The chromatographic column has the advantages of small particle size, short length, high separation potency and high efficiency. After gradient modification of the mobile phase, CTS and LTD exhibited a relatively long retention time, a good peak shape and a high degree of separation when the methanol-1% formic acid water ratio was 90:10 (V/V). The CTS and LTD response and separation were better when the injection volume was 5 μL. Furthermore, spray voltage, capillary temperature, sheath gas and auxiliary gas were optimized for CTS and LTD standard solutions to improve the charged rate of the compounds. The CTS and LTD were completely decomposed into stable daughter ions by optimizing collision energy and collision gas pressure. Appropriate collision conditions and daughter ions were selected respectively to conduct quantitative analysis on ion pairs.

### 3.3 Validation of the HPLC-MS/MS method for simultaneous quantitative analysis of CTS

The CTS and INTERNAL standard LTD specific chromatograms obtained from blank plasma samples, low-concentration CTS quality control samples, and 60 mg/kg CTS after oral administration were detected. The peak times of CTS and internal standard LTD were about 1.54 and 1.38 min, respectively. There was no interference with each other and no interference from endogenous substances. It demonstrated that the analytical method is specific.

The linear range of CTS concentration in plasma, heart, spleen, kidney, and brain samples was 1–200 ng/mL, whereas the linear range of CTS concentration in liver and lung samples was 2–500 ng/mL. Analysis of plasma quality control samples from the same batch demonstrated RE of −9.2% to −3.0% and RSD of 2.7%–8.6%. Plasma quality control samples were analyzed for three consecutive days with RE of −5.8% to −1.5% and RSD of 4.0%–7.2%. Additionally, the RE of the same batch and the quality control samples of each organization for three consecutive days were all in the range of −20% to 20%, with RSD of less than 20%, all in line with the guidelines. According to the HPLC-MS/MS analysis established in this study, the lower limit of quantification of CTS in plasma samples was 1 ng/mL. The lower limit of quantification was 1 ng/mL in heart, spleen, kidney, and brain samples, while 2 ng/mL in liver and lung samples. The RE and RSD of six LLOQ samples in plasma were 0.0% and 7.1% respectively. The RE of LLOQ samples in each tissue was in the range of −20% to 20%, with RSD less than 20%.

The results demonstrated that the experiments were consistent and reproducible, that the method provided sufficient exclusivity, and that the HPLC-MS/MS method was sensitive and efficient enough to be used for routine analysis and pharmacokinetic studies of analyses.

### 3.4 Pharmacokinetic study

Normal rats and pulmonary fibrosis models were given a single dose of 60 mg/kg CTS on days 14 and 28, and normal rats and pulmonary fibrosis models were given a continuous dose of 60 mg/kg CTS for 28 days. The validated assay was successfully applied to both groups. Simultaneously, the concentration data of plasma CTS at different time points were measured for pharmacokinetic study. The mean plasma concentration-time curves based on the data and the major pharmacokinetic parameters calculated based on the non-AV model are provided in the Figure/Table.

**FIGURE 2:**
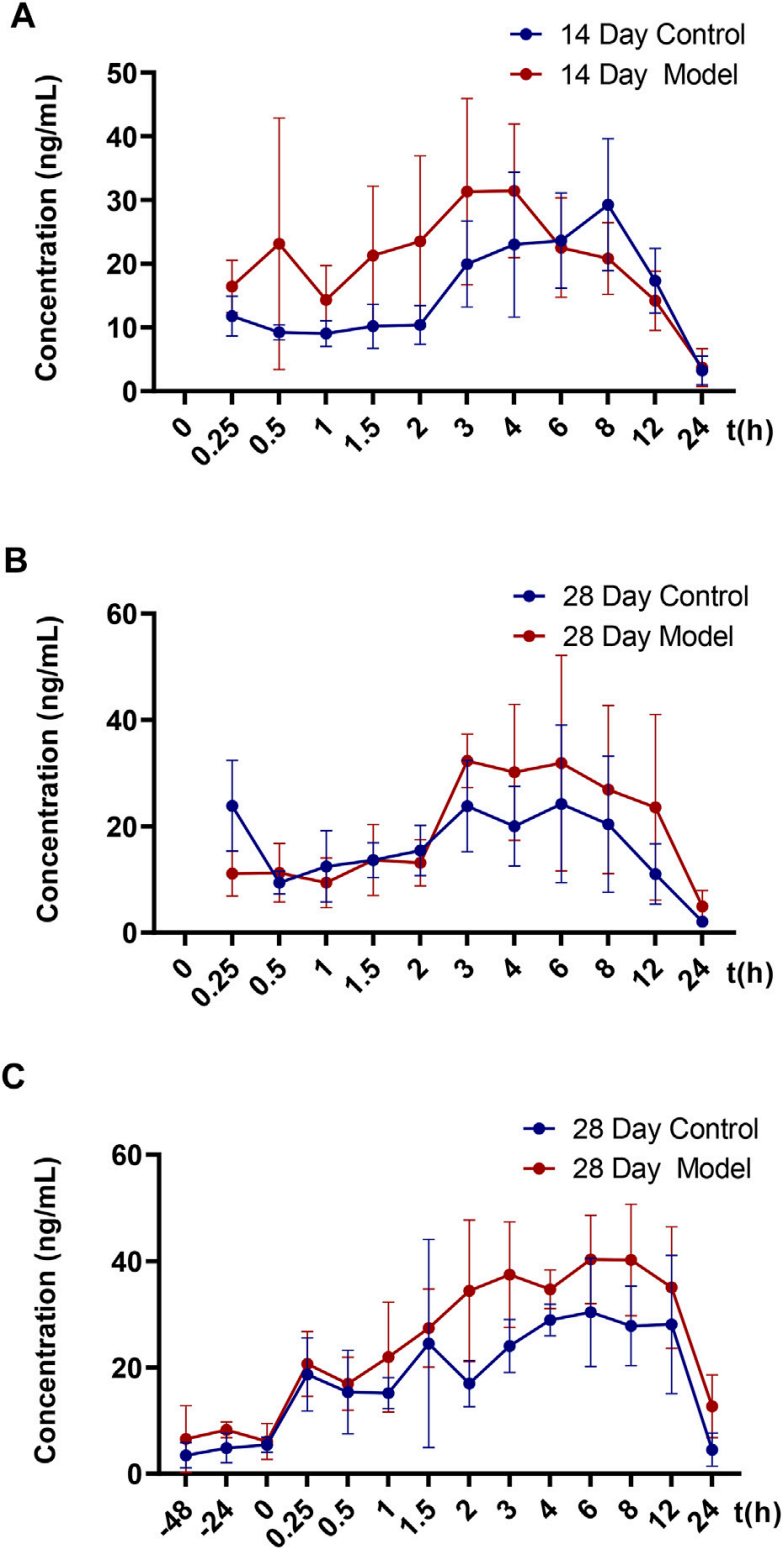
Mean blood concentration-time curve of CTS in SD rats. **(A)** Mean plasma concentration-time curves of 14-day single dose CTS in control and model SD rats (*n* = 5). **(B)** Mean plasma concentration-time curves of 28-day single dose CTS in control and model SD rats (*n* = 5). **(C)** Mean plasma concentration-time curves of 28-day multi-dose CTS in control and model SD rats (*n* = 5).

Figure 2 and Table 2 illustrate the 14-day and 28-day data of CTS single dose, whereas Figure 2 and Table 3 depict the 28-day data of multiple doses.

**TABLE 2:** Major pharmacokinetic parameters of rats in the single-dose control group and model group on days 14 and 28 (mean ± SD, *n* = 5, *p < 0.05 *versus* Day 28 Control group).

	Day 14		Day 28	
PK parameters	Control	Model	Control	Model
T_max_ (h)	6.40 ± 2.19	4.00 ± 1.22	5.00 ± 1.41	4.60 ± 2.30
C_max_ (ng/mL)	32.23 ± 6.65	41.52 ± 10.79	32.79 ± 10.57	40.80 ± 15.48
AUC_0-t_ (ng/mL·h)	325.9 ± 140.8	366.8 ± 58.8	297.8 ± 137.9	467.2 ± 258.1
AUC_0-∞_ (ng/mL·h)	426.9 ± 99.9	385.8 ± 70.7	310.4 ± 145.7	518.1 ± 276.8
CL/F (L/h)	147.5 ± 37.2	160.1 ± 31.6	220.0 ± 74.4	146.1 ± 74.2
V_d_/F (L)	1408.3 ± 634.5	1139.3 ± 152.7	1441.7 ± 417.5	1570.7 ± 1067.2
t_1/2_ (h)	6.88 ± 3.22	5.02 ± 0.81	4.63 ± 0.59	7.03 ± 1.20*
MRT (h)	12.83 ± 4.43	9.42 ± 1.37	8.85 ± 0.63	11.13 ± 0.99*

**TABLE 3:** Major pharmacokinetic parameters of 28-day multi-dose control group and model group (Mean ± SD, *n* = 5, *p < 0.05 *versus* Control group).

	Day 28	
Parameter	Control	Model
T_max_ (h)	7.40 ± 4.34	8.00 ± 2.45
C_max_ (ng/mL)	39.30 ± 14.31	45.10 ± 7.42
AUC_0-t_ (ng/mL·h)	507.0 ± 152.2	709.6 ± 143.8
AUC_0-∞_ (ng/mL·h)	567.0 ± 178.2	988.4 ± 318.8*
CL/F (L/h)	113.6 ± 31.5	65.8 ± 21.0*
V_d_/F (L)	1128.5 ± 285.7	1081.1 ± 394.8
t_1/2_ (h)	7.11 ± 1.86	12.71 ± 8.01
MRT (h)	10.95 ± 2.77	19.40 ± 10.48
C_av(ss)_ (ng/mL)	4.14 ± 1.74	7.41 ± 3.08
DF	9.18 ± 2.52	6.03 ± 2.69
AUC_ss_ (ng/mL·h)	99.3 ± 41.74	177.9 ± 73.9

The results of the *t*-test demonstrated no significant differences in major pharmacokinetic parameters between the control and the model groups at 14 days of a single dose (*p* < 0.05). Based on the average blood concentration-time curve, the model group exhibited faster absorption and an earlier peak time. The control group and model groups indicated a bimodal phenomenon with low oral bioavailability. After a single dose of 28 days, the elimination half-life and mean dwell time were significantly different between the control and the model groups (*p* < 0.05), but there was no significant difference in other parameters (*p* < 0.05). The elimination half-life and average dwell time of the model group were significantly longer than those of the control group. The area under the curve was larger than that in the control group, but there was no statistical difference between individuals. The control group rats and the model group rats also exhibited a bimodal phenomenon.

After 28 days of continuous administration, there were significant differences in the curve area and clearance rate between the control and the model groups (*p* < 0.05), and there was no significant difference in other parameters (*p* > 0.05). The elimination half-life, average dwell time, average steady-state concentration and steady-state drug administration curve area of the model group were also significantly increased compared to the control rats, but there was no statistical difference due to the large individual differences. The blood concentration values demonstrated that the three steady-state blood concentrations were all low.

The model group had a higher blood concentration than the control group at each time point. At the last blood collection point, the plasma concentration of CTS in the model group was still higher. The average blood concentration-time curve observation revealed that the bimodal phenomenon also exists in multi-dose administration. Additionally, the individual differences of model group rats were significantly greater than those of control group rats.

### 3.5 Tissue distribution study

**FIGURE 3:**
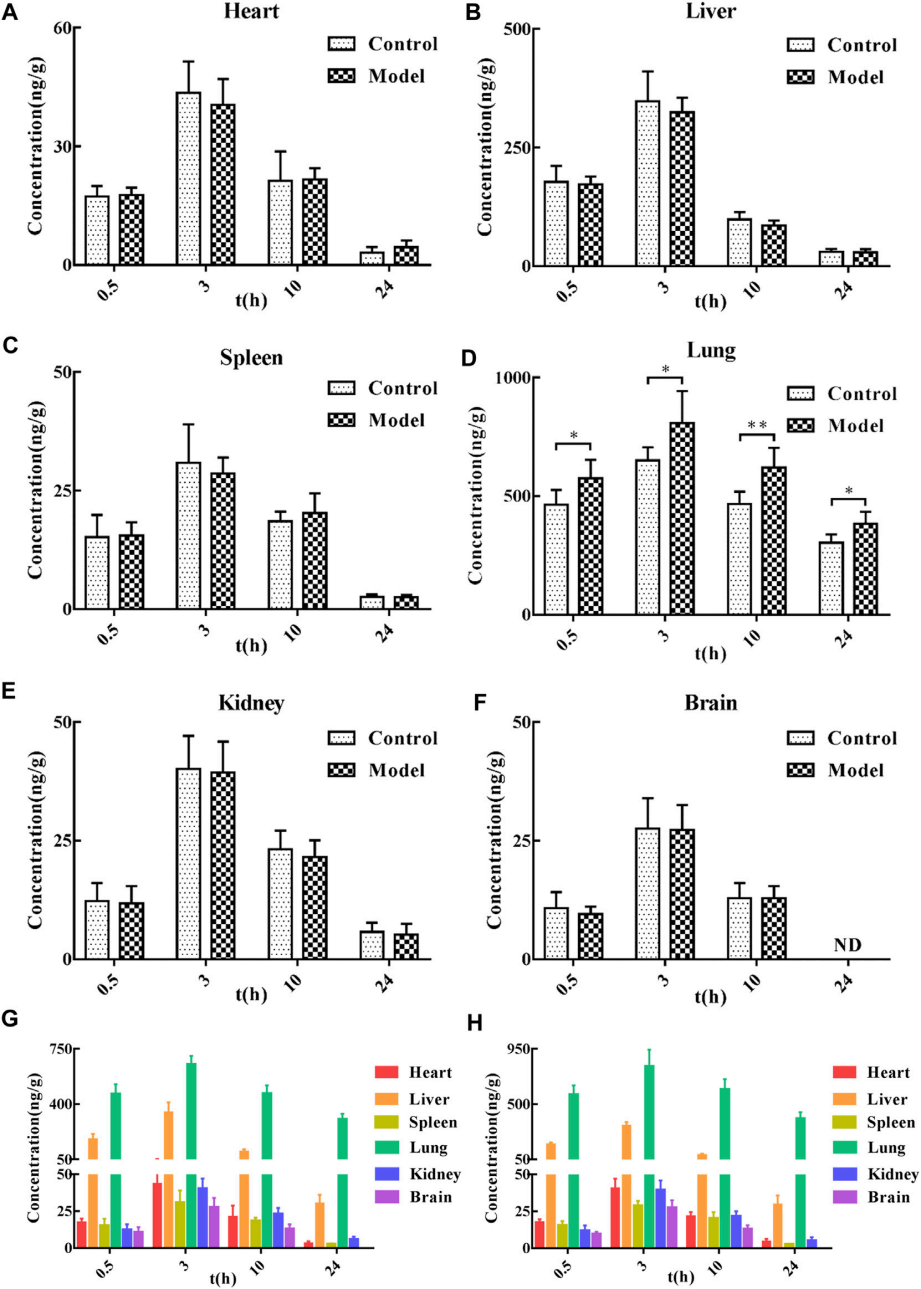
Tissue distribution of CTS in SD rats. **(A–F)** The concentration of SD rats in the control group and model group at different time points after oral administration of 60 mg/kg CTS. **(G)** The concentration of SD rats in the control group at different time points after oral administration of 60 mg/kg CTS. **(H)** The concentrations of 60 mg/kg CTS in each tissue at different time points in the model group. One-way analysis of variance combined with Dunnett’s multiple comparison method was used for analysis, and all data were expressed as mean ± SD. *p < 0.05; **p < 0.01.

After a single oral administration of 60 mg/kg CTS, the concentrations of CTS in various tissues (heart, liver, spleen, lung, kidney, and brain) at different time points were measured (Figure 3). CTS was widely distributed in various organs, including the lung and liver. In the control and the model groups, the distribution trend of CTS was consistent: lung > liver > heart > spleen > kidney > brain at 0.5 h, lung > liver > heart > kidney > spleen > brain at 3 h, lung > liver > heart ≈ kidney > spleen > brain at 10 h, lung > liver > kidney > heart > spleen > brain at 24 h. The amount of CTS exposure in the heart, liver, spleen, kidney, and brain of the two groups of rats did not differ significantly. However, the exposure amount in the lungs of the model group was significantly higher than that in the control group.

## 4 Discussion

Pharmacokinetic research is an important part of the process of new drug development, because it guides the entire process and serves as a foundation for pharmacodynamics, toxicology and drug preparation research. Pharmacokinetic studies in animal models of disease can better reflect the dynamic changes of drugs and explain the pharmacokinetic basis of the pharmacological effects of drugs. BLM is a basic glycopeptide anticancer antibiotic. It is frequently used as an intratracheal infusion in experimental animals to cause severe inflammation and pulmonary fibrosis (Della Latta et al., 2015) due to its strong pulmonary toxicity (Moeller et al., 2008). In the previous pharmacodynamic study of CTS in pulmonary fibrosis rats treatment, the high-dose group of 60 mg/kg exhibited a good pharmacodynamic effect and no side effects (Zhang et al., 2019). Additionally, the amount of oral CTS inhaled into the systemic circulation was small (Song et al., 2007; Wang et al., 2020), so choosing a higher dose may be beneficial to compare the pharmacokinetic characteristics of CTS in normal and pulmonary fibrosis rats.

After intragastric administration of CTS to rats, the absorption was slow, the amount absorbed into the systemic circulation was low, the CTS was widely distributed in the body, and the elimination rate was relatively slow. At 14 days, the pharmacokinetic characteristics of the control and the model groups were the same. The faster absorption of the rats in the model group may be due to the increased permeability of vascular endothelial cells caused by the inflammatory reaction (Wautier and Wautier, 2022). The 28-day model group had slower elimination and longer dwell time, and the area under the curve was significantly higher than in the control group. This may be due to the decline of lung function or even the overall physiological function of rats; the speed of CTS clearance metabolism is reduced. It is also possible that the drug accumulation in the substantively diseased lungs increased, and CTS released slowly from the lungs into the blood. Changes in intestinal microbiota under pathological conditions may lead to slower metabolic rates (Morgan et al., 2018; Parvez et al., 2021). However, the steady-state blood concentration and the area under the curve of the model group were slightly higher than those of the control group, and the area under the curve and clearance rate of the model group were significantly higher than those of the control group, which was further amplified by long-term administration. These results suggest that in rats with advanced pulmonary fibrosis, the substantial lung lesions may greatly influence the pharmacokinetic behavior of CTS, resulting in the slow elimination of CTS. Moreover, a bimodal phenomenon occurred in all experimental groups, a common phenomenon in non-intravenous drug injection, possibly because CTS is widely distributed *in vivo* and tissue redistribution occurs. Gastrointestinal imbalance (Hatton et al., 2019) and liver-intestinal circulation also caused the bimodal phenomenon.

The tissue distribution study revealed that CTS could be detected in all tissues 0.5 h after administration of 60 mg/kg CTS administration by gavage, indicating that CTS could be rapidly and widely distributed in various tissues and organs after oral administration. The peak concentration of all tissues reached 3 h after administration, and high concentrations of CTS could still be detected at 10 h after administration. CTS could be detected at 24 h after administration except in brain tissue, indicating that CTS was retained in all tissue and organs for a long time. After gavage of CTS to rats, the concentration of CTS in lungs and liver was higher than in other tissues and organs, indicating that they are likely to be the effector organs or toxic organs of CTS. This result is consistent with literature reports: CTS has therapeutic effects in liver cancer, ethanol-induced liver injury (Nagappan et al., 2019), liver failure (Jin et al., 2014), liver fibrosis (Han et al., 2019), lung cancer (Vundavilli et al., 2020) and pulmonary fibrosis (Zhang et al., 2020), among other diseases (Liu et al., 2021), and is metabolized through in the liver (Zeng et al., 2018). Compared to the control group, there was no significant difference in the amount of CTS exposure in the heart, liver, spleen, kidney, and brain of rats in the model group after the gavage of CTS. However, the amount of CTS exposure in the lung tissue was significantly increased, indicating that the pathological changes of pulmonary fibrosis are conducive to the targeted distribution of CTS in the lung. This phenomenon may be caused by pulmonary vascular hyperplasia accompanied by increased permeability of vascular endothelial cells. It is also possible that components of lung dysplasia (collagen, collagen fibers, aminoglycans, or cytokines) have a better binding effect on CTS.

## 5 Conclusion

This was the first study to investigate the pharmacokinetic characteristics and tissue distribution of CTS in normal and pulmonary fibrosis rats, and to compare the pharmacokinetic and tissue distribution differences between the two rat groups. The results demonstrated that the distribution and metabolism of CTS and the targeted distribution of CTS in the lungs were affected by the pathological conditions of pulmonary fibrosis, as evidenced by the increase of the area under the curve, clearance half-life, average dwell time and high exposure of CTS in the lungs of pulmonary fibrosis rats.

## Data Availability

The original contributions presented in the study are included in the article/Supplementary Material, further inquiries can be directed to the corresponding authors.
