# Supplementary material for: Pharmacokinetics and tissue distribution of bleomycin-induced idiopathic pulmonary fibrosis rats treated with cryptotanshinone
Source: Front Pharmacol. 2023 Mar 9;14:1127219. doi: 10.3389/fphar.2023.1127219 (PMC10034131; doi:10.3389/fphar.2023.1127219)
Supplement: Supplementary file 1 [file DataSheet1.PDF]

1. specificity

The optimized HPLC-MS/MS analysis method was used to detect blank plasma samples, low-concentration cryptotanshinone quality control samples, and biological samples after gavage of 60 mg/kg cryptotanshinone. Specific chromatograms of CTS and internal standard LTD were obtained, as shown in Fig. 1 and Fig. 2. The peak time of CTS and internal standard LTD was about 1.54 min and 1.38 min respectively, and there was no interference between each other, and no interference from internal substances, indicating that the proposed analytical method was highly specific.

(A)

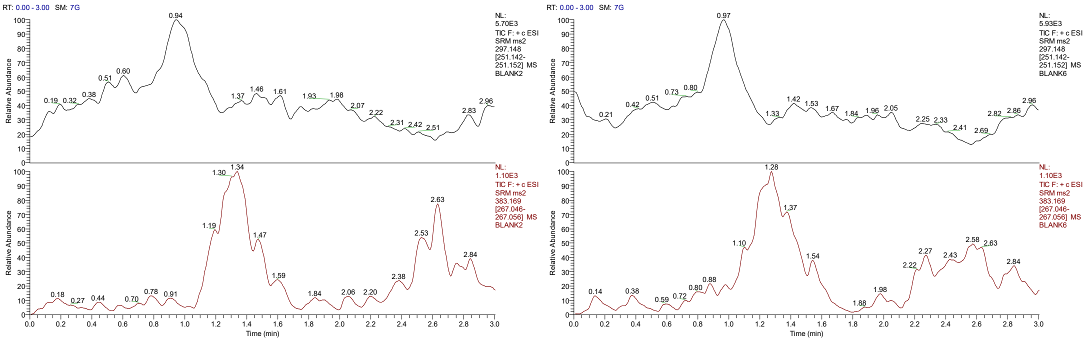

(B)

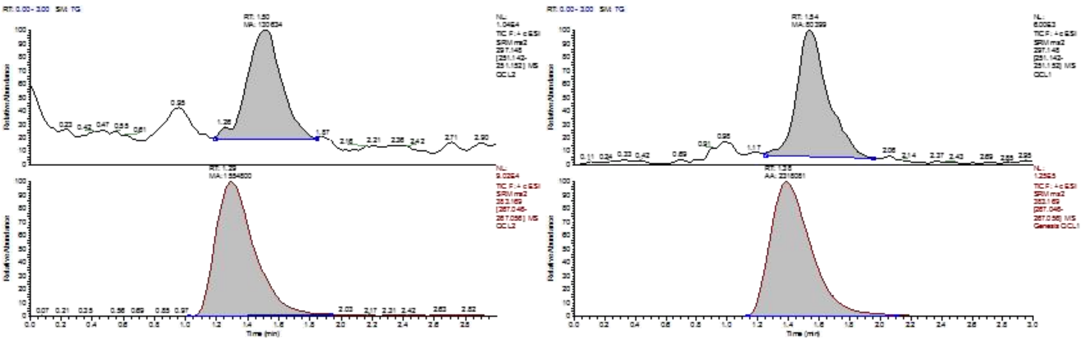

(C)

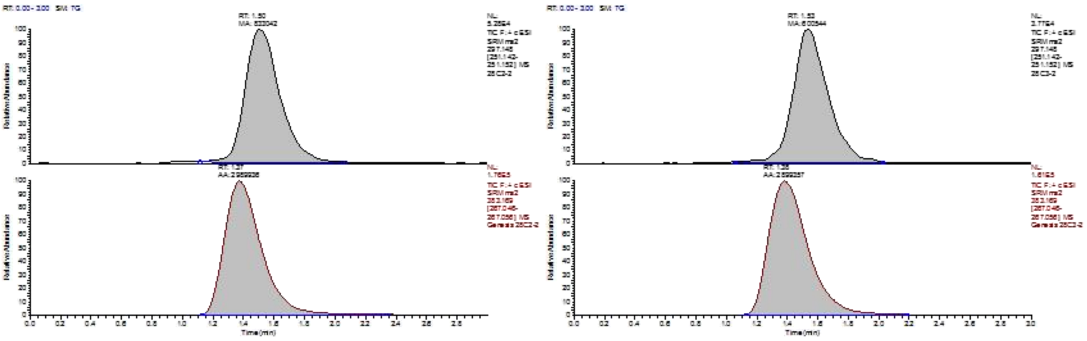

Fig. 1 The Chromatograms of CTS and LTD in rat plasma

A: Samples of rat blank plasma; B: Low concentration quality control samples of CTS; C: Samples of rat plasma collected at 15 min after intragastric administration of CTS.

A1

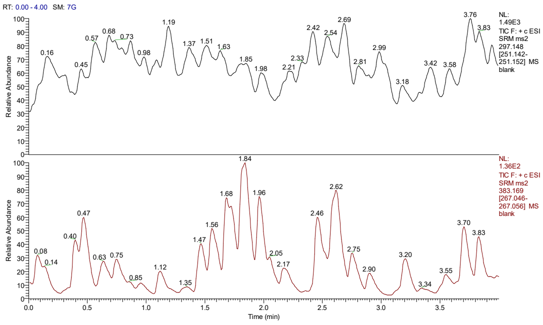

A2

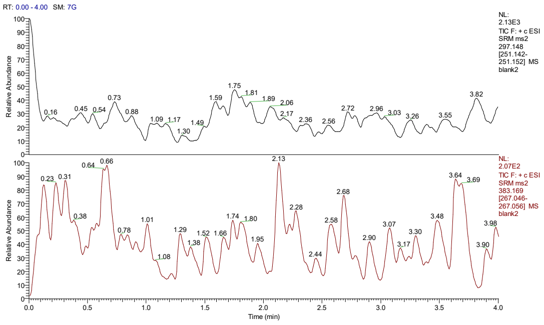

A3

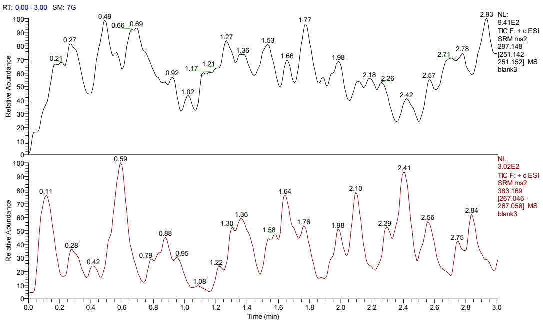

A4

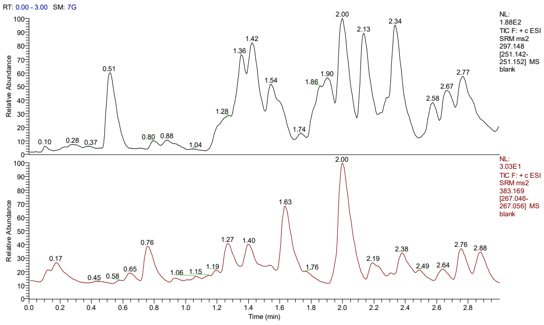

A5

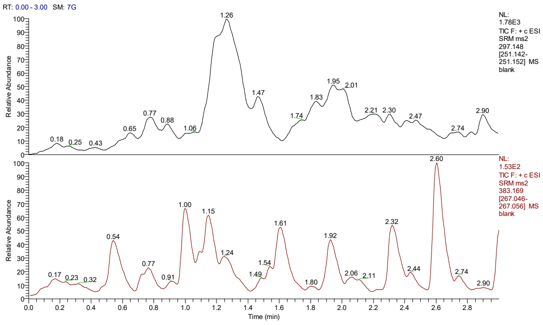

A6

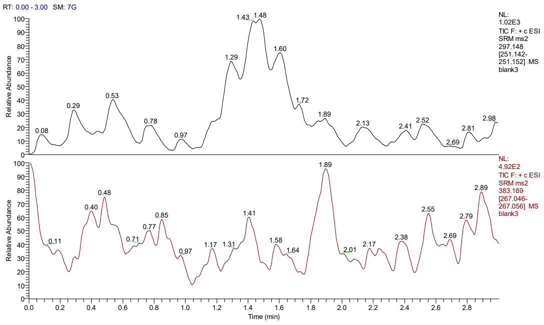

B1

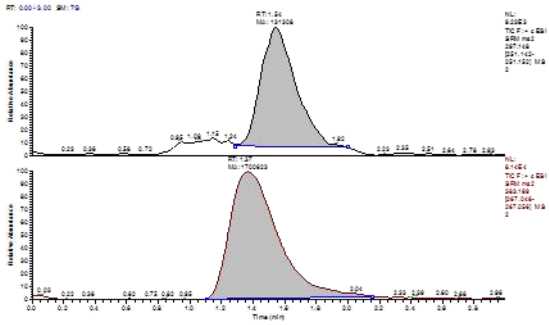

B2

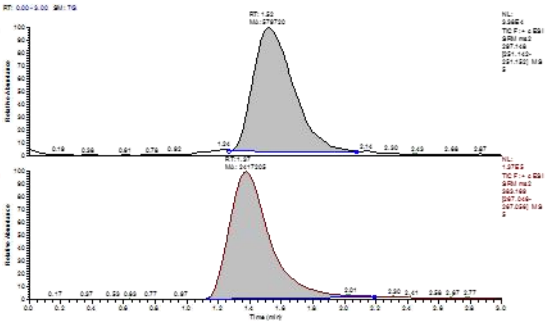

B3

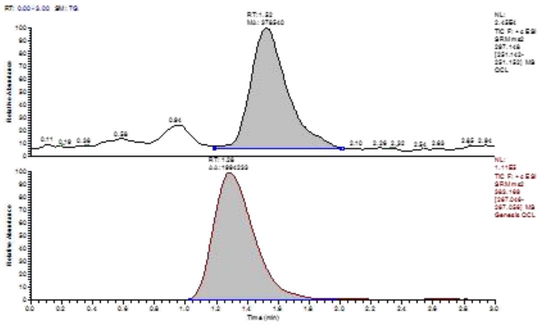

B4

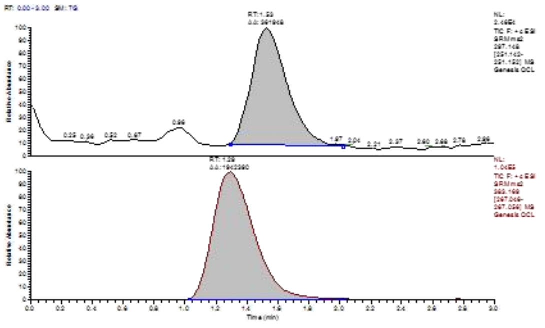

B5

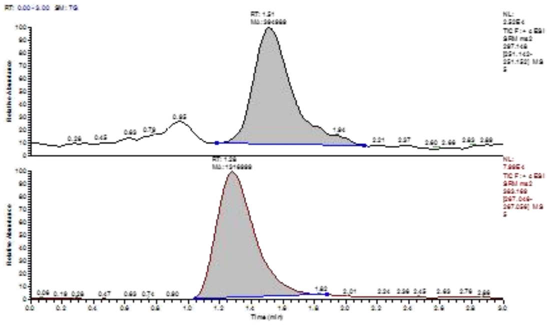

B6

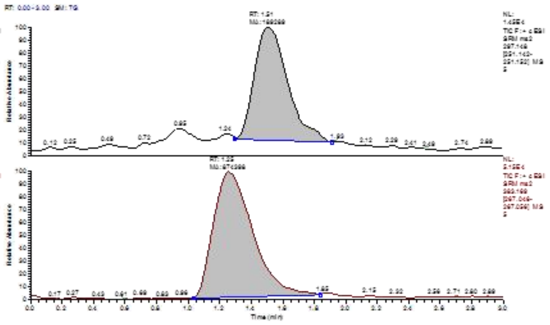

C1

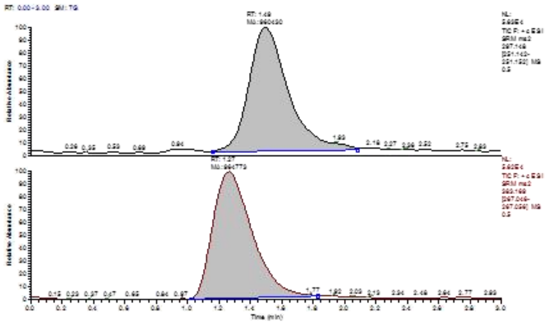

C2

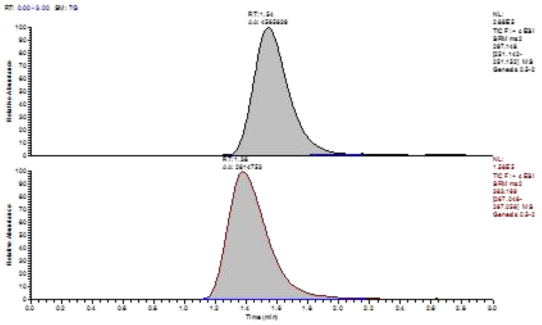

C3

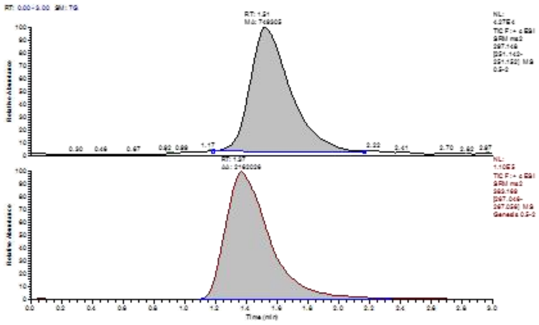

C4

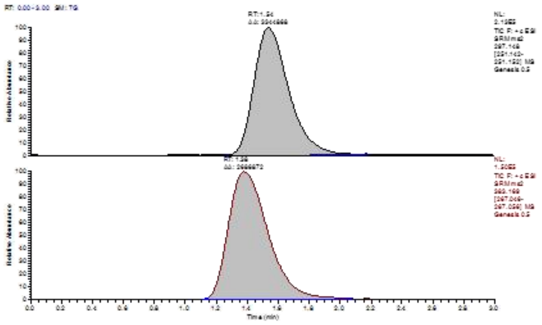

C5

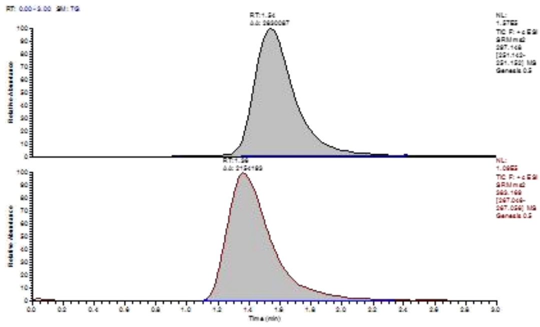

C6

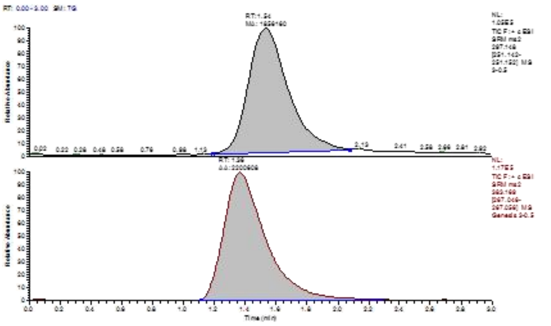

Fig. 2 The Chromatograms of CTS and LTD in rat tissue samples

A: Samples of rat blank tissue; B: Low concentration quality control samples of CTS; C: Samples of rat tissue collected at 0.5 h after intragastric administration of CTS.

1: Heart; 2: Liver; 3: Spleen; 4: Lung; 5: Kidney; 6: Brain.

2 Linearity range

With the concentration of CTS as the horizontal coordinate and the ratio of peak area of CTS to peak area of internal standard LTD as the vertical coordinate, the standard curve of plasma and tissue samples was obtained. The linear range of plasma, heart, spleen, kidney and brain was 1-200 ng/mL, and that of liver and lung was 2-500 ng/mL. According to the results of linear regression analysis, the regression equation and correlation coefficient of standard curve were obtained. The regression equation of standard curve accompanying multiple analysis batches of plasma samples was shown in Table 1, and the standard curve of each tissue was shown in Table 2. The results showed that the ratio of peak area of CTS to peak area of internal standard LTD had a linear relationship with CTS concentration in the linear range, and the correlation coefficient was good. The linear range of CTS concentration in plasma, heart, spleen, kidney and brain samples was 1-200 ng/mL, and the linear range of CTS concentration in liver and lung samples was 2-500 ng/mL.

Tab. 1 Regression equation of CTS standard curve in rat plasma (Weight: 1/X)

Number	Regression equation	Correlation coefficient (R^2)	Liner range (ng/mL)
1	Y=0.0109776+0.0292387*X	0.9997	1-200
2	Y=0.00681402+0.0481686*X	0.9997	1-200
3	Y=0.0336354+0.0260112*X	0.9980	1-200
4	Y=0.033583+0.0175219*X	0.9963	1-200
5	Y=0.0145648+0.021272*X	0.9992	1-200
6	Y=0.0654197+0.034663*X	0.9992	1-200
7	Y=0.0782579+0.039431*X	0.9981	1-200
8	Y=0.0300691+0.041431*X	0.9997	1-200

Tab. 2 Regression equation of CTS standard curve in rat tissue (Weight: 1/X)

Tissue	Regression equation	Correlation coefficient (R^2)	Liner range (ng/mL)
Heart	Y=0.0710701+0.0398670*X	0.9996	1-200
Liver	Y=0.0386992+0.0220133*X	0.9986	2-500
Spleen	Y=0.0884906+0.0364942*X	0.9997	1-200
Lung	Y=0.0397427+0.0225064*X	0.9996	2-500
Kidney	Y=0.0851299+0.0498844*X	0.9975	1-200
Brain	Y=0.0952980+0.0454726*X	0.9973	1-200

3. LLOQ

The lower limit of quantification of CTS in plasma samples was 1 ng/mL, and the confirmed results of the lower limit of quantification were shown in Table 3. The lower limit of quantification was 1 ng/mL for heart, spleen, kidney and brain samples, and 2 ng/mL for liver and lung samples. The confirmed results were shown in Table 4. The results showed that the RE and RSDS of 6 LLOQ samples were 0.0% and 7.1% respectively. RE of LLOQ samples in all tissues was in the range of -20%-20%,

RSDS were all less than 20%, and the results were in line with the guidelines and met the requirements of analysis.

Tab. 3 LLOQ, intra-batch precision and accuracy of CTS in rat plasma

	Number	Concentration（ng/mL）			
		1.0	2.0	20.0	200.0
Measured concentration（ng/mL）	1	0.9	1.8	19.0	177.6
	2	1.0	1.9	18.2	177.1
	3	1.1	2.2	17.6	180.0
	4	1.0	1.8	18.8	188.3
	5	1.0	2.0	20.2	185.3
	6	1.1			
Mean		1.0	1.9	18.8	181.7
SD		0.1	0.2	1.0	4.9
RSD（%）		7.1	8.6	5.2	2.7
RE（%）		0.0	-3.0	-6.2	-9.2

Tab. 4 LLOQ of CTS in rat tissue samples（n=6）

Tissue	Concentration（ng/mL）	Measured con.（ng/mL）		*RSD*（%）	*RE*（%）
		Mean	*SD*		
Heart	1	1.1	0.1	10.1	6.7
Liver	2	2.0	0.2	9.0	-1.8
Spleen	1	1.0	0.1	9.8	2.2
Lung	2	2.1	0.1	3.5	5.4
Kidney	1	1.1	0.1	11.9	12.9
Brain	1	1.0	0.1	7.8	-2.2

4. In - batch precision accuracy

The analysis of plasma quality control samples from the same batch showed that

RE and RSDS of low concentration plasma quality control samples were -3.0% and 8.6% respectively. The deviation of plasma concentration control samples was -6.2% and RSDS were 5.2%. The plasma concentration of RE was -9.2% and RSDS were 2.7%, as shown in Table 2-4. For the tissue quality control samples of the same batch, RE of low, medium and high concentration results were all within the range of -20%-20%, RSDS were all less than 20%, as shown in Table 5. The results were in line with the guidelines and met the analysis requirements.

Tab. 5 Intra-batch precision and accuracy of CTS in rat tissue samples （*n*=5）

Tissue	Concentration （ng/mL）	Measured con. （ng/mL）		*RSD*（%）	*RE*（%）
		Mean	*SD*		
Heart	2	2.1	0.1	6.7	7.1
	20	19.9	2.0	10.0	-0.6
	200	182.1	14.3	7.8	-9.0
Liver	5	5.2	0.4	6.8	4.1
	50	48.4	2.8	5.9	-3.3
	300	286.0	12.1	4.2	-4.7
Spleen	2	2.2	0.1	6.6	8.8
	20	19.4	1.2	6.2	-3.1
	200	196.3	6.1	3.1	-1.8
Lung	5	5.3	0.1	2.3	6.2
	50	49.1	1.3	2.7	-1.7
	300	281.1	7.1	2.5	-6.3
Kidney	2	2.2	0.1	5.1	9.2
	20	19.8	0.6	2.9	-0.9
	200	188.9	1.8	1.0	-5.5
Brain	2	2.2	0.1	6.8	8.4
	20	20.7	1.5	7.0	3.3
	200	184.7	7.3	4.0	-7.6

5 Precision accuracy between batches

The analysis of plasma quality control samples for three consecutive days showed that RE and RSDS of low-concentration quality control samples were -1.5% and 7.2%. RE and RSDS of medium concentration control samples were -5.8% and 4.0% respectively. High concentration RE was -5.5% and RSDS were 4.3%. The results were shown in Table 6. For the tissue quality control samples for three consecutive days, RE of low, medium and high concentration results were all within the range of -20%-20%, RSDS were all less than 20%, which were in line with the guidelines. The results are shown in Table 7.

Tab. 6 Inter-batch precision and accuracy of CTS in rat plasma

Batch number	Number	Concentration（ng/mL）		
		2.0	20.0	200.0
1	1	1.8	19.0	177.6
	2	1.9	18.2	177.1
	3	2.2	17.6	180.0
	4	1.8	18.8	188.3
	5	2.0	20.2	185.3
2	1	2.0	19.1	187.8
	2	2.1	18.3	192.2
	3	2.1	18.6	178.4
	4	2.2	19.1	182.6
	5	1.9	18.2	188.5
3	1	1.7	20.5	180.6
	2	1.8	18.7	205.7
	3	1.9	18.8	188.7
	4	2.0	19.2	198.3
	5	2.1	17.8	186.5
Mean		2.0	18.8	188.9
SD		0.1	0.7	8.2

RSD（%）	7.2	4.0	4.3
RE（%）	-1.5	-5.8	-5.5

Tab. 7　Inter-batch precision and accuracy of CTS in rat tissue samples（*n*=5*3）

Tissue	Concentration（ng/mL）	Measured con.（ng/mL）		*RSD*（%）	*RE*（%）
		Mean	*SD*		
Heart	2	2.0	0.2	8.9	-0.9
	20	21.1	2.4	11.5	5.3
	200	189.5	10.7	5.6	-5.3
Liver	5	5.1	0.2	4.8	1.4
	50	48.2	3.3	6.9	-3.6
	300	283.0	7.6	2.7	-5.7
Spleen	2	2.2	0.1	6.1	12.0
	20	20.3	1.2	6.1	1.5
	200	189.2	9.7	5.1	-5.4
Lung	5	5.2	0.2	3.3	4.1
	50	50.5	2.1	4.1	0.9
	300	285.7	9.2	3.2	-4.8
Kidney	2	2.2	0.1	6.0	9.6
	20	19.5	0.8	4.0	-2.4
	200	187.5	6.4	3.4	-6.2
Brain	2	2.1	0.2	7.9	6.8
	20	19.5	0.9	4.7	-2.3
	200	190.7	6.4	3.4	-4.7
